# Body Size Measurements Grouped Independently of Common Clinical Measures of Metabolic Health: An Exploratory Factor Analysis

**DOI:** 10.3390/nu16172874

**Published:** 2024-08-27

**Authors:** Katie M. Ellison, Aseel El Zein, Chelsi Reynolds, Sarah E. Ehrlicher, Julianne G. Clina, Tsz-Kiu Chui, Kimberly A. Smith, James O. Hill, Holly R. Wyatt, R. Drew Sayer

**Affiliations:** 1 Department of Nutrition Sciences, University of Alabama at Birmingham, Birmingham, AL 35233, USA; vidya25@uab.edu (K.M.E.); hillj@uab.edu (J.O.H.); drholly@uab.edu (H.R.W.); 2 Department of Family and Community Medicine, University of Alabama at Birmingham, Birmingham, AL 35294, USA; 3 Department of Internal Medicine, University of Kansas Medical Center, Kansas City, MO 66103, USA

**Keywords:** cardiometabolic disease, obesity, exploratory factor analysis, body positivity

## Abstract

Background: Obesity is commonly aggregated with indices of metabolic health. Proponents of body positivity approaches question whether body size is a determinant of health and well-being. Our objective was to conduct an exploratory factor analysis (EFA) to determine if body size measurements factor load with or independent of metabolic health measures. Methods: The EFA was conducted on *n*= 249 adults using baseline data from four weight loss trials (Sample 1: *n* = 40; Sample 2: *n* = 52; Sample 3: *n* = 53; Sample 4: *n* = 104). An EFA of nine items (systolic blood pressure [SBP], diastolic blood pressure [DBP], hemoglobin A1c [HbA1c], HDL-cholesterol [HDL], LDL-cholesterol [LDL], total cholesterol [TC], body mass index [BMI], body fat percent BF%], and waist circumference [WC]) was conducted with oblique rotation. Results: Three factors were retained, which produced a model explaining 87.5% of the variance. Six items loaded strongly (>0.8) under three components and were selected for retention (Factor 1: LDL and TC; Factor 2: BMI and WC; Factor 3: SBP and DBP). Conclusion: Body size measures loaded separately from measures of metabolic health and metabolic health were further split into lipid- and blood pressure-focused factors. These results support weight-neutral interventions to improve overall health and well-being.

## 1. Introduction

One of the hallmarks of obesity is that it increases risk of developing cardiometabolic diseases (CMD), especially type 2 diabetes (T2D) and cardiovascular disease (CVD). Altogether, CMDs were responsible for the deaths of over 4.8 million adults in the United States between 1990 and 2017 [1]. Intentional weight loss has traditionally been the first line treatment approach for reducing CMD risk in people with obesity because of its well-documented metabolic benefits [2,3]. Reducing body weight by 5–10% can yield clinically meaningful improvements in CMD risk factors, and this range of weight loss has become a widely accepted benchmark for “successful” weight loss by obesity clinicians and researchers [3,4]. Despite the established importance of weight loss to reduce the metabolic disease risk associated with obesity, proponents of weight-neutral and body positivity approaches raise questions about the utility of focusing on body weight [5,6]. It is suggested that body size is not the only determinant of health and well-being and that weight loss should be optional for people with obesity to be healthy [7].

Weight neutral approaches to obesity treatment such as Health at Every Size^®^ (HAES, trademarked by the Association for Size Diversity and Health) encourages people of all body sizes to engage in behaviors such as self-acceptance, intuitive eating, and physical activity instead of emphasizing weight loss [8]. The paradigm also encourages deemphasizing the measure of body mass index (BMI) so that healthcare decisions are not based on body size [5]. These principles contradict traditional obesity treatment approaches and have sparked a provocative debate among the scientific community. Critics are quick to point out that weight loss is effective for preventing and managing several chronic diseases and believe that individuals have a personal responsibility to manage their health and weight [9]. HAES^®^ supporters argue that the presumption that people with obesity have failed to take individual responsibility has led to “fatphobia” (anti-fat bias) and neglects to consider the physiological and environmental factors associated with the etiology of obesity [9].

From a critic’s perspective, an extensive body of research supports weight loss as an effective means for treating obesity-related CMD [3,10]. Moreover, a clinician may prescribe a treatment regimen (e.g., calorie restriction for weight loss), but it is up to the individual to determine if they accept the responsibility of behavior change. This reasoning suggests that long-term weight loss is achievable through behavior change if the person is determined to do so. From a weight-neutral and body positivity perspective, the traditional intervention approach of “eat less, move more” has not been effective in producing long-term results or curtailing obesity rates [11,12,13]. Also, research shows that the development and expression of obesity is multifactorial and characterized by a combination of physiological, behavioral, and environmental factors [3]. This reasoning suggests traditional obesity treatment is flawed, possibly because it is not as diverse as the factors that contribute to how obesity is developed and expressed. Considering both sides of this debate, it may be time to identify treatment modalities that do not solely focus on body size but rather expand the metrics of successful treatment to more holistic measures of health and well-being as suggested by HAES^®^ and similar approaches.

While conceptual debate is critical to transform understanding into real-world solutions, more empirical evidence is necessary to move the debate forward. Here, we take a data-driven approach to investigate the construct(s) driving obesity and CMD. The purpose of this research is to conduct an exploratory factor analysis (EFA) to determine if measurements of body size covary with or independent of common clinical measures of cardiometabolic health to identify potential latent constructs. If these constructs are independent, further investigation into holistic obesity treatments (with or without weight loss) may be warranted. We hypothesize that obesity and CMD are multidimensional and that the EFA will reveal two distinct factors: metabolic health and body size.

## 2. Materials and Methods

### 2.1. Subjects

Baseline data from a convenience sample of 250 adults from four behavioral weight loss interventions were used for analyses. Putative measures were performed at baseline study visits, which took approximately 60 min to administer. Participants were instructed to fast for at least 8 h and stay hydrated while refraining from moderate-to-vigorous physical activity for 24 h prior to testing. Descriptions of each study can be found in Table 1.

### 2.2. Demographics

Demographic information was collected via REDCap 14.6.1 and is presented in Table 2. These data were collected at baseline through a questionnaire. The EFA included nine items of two wellness dimensions and are described below. Metabolic data were collected from either laboratory-drawn blood samples or a fingerstick blood sample analyzed by point of care (POC) devices.

### 2.3. Metabolic Health Measures

Laboratory-drawn blood samples and analyses: Metabolic data from trials NCT04392284 and NCT03832933 were obtained using laboratory-drawn blood samples. Fasting concentrations of total cholesterol (TC), high-density lipoprotein cholesterol (HDL-C), and low-density lipoprotein cholesterol (LDL-C) were drawn at the Laboratory of the Family and Community Medicine Clinic or Encompass Health Lakeshore Rehabilitation Hospital and transported to the CLIA-certified analytic laboratory managed by the UAB Department of Pathology (Outreach Lab, Birmingham, AL, USA).

POC fingerstick samples and analyses: Metabolic data from trials NCT04014296 and NCT04745572 were obtained from POC devices. Participants were instructed to rest for five minutes and then 40 mcg of blood was filled into a capillary tube from the tip of the ring finger of their non-dominant hand. A plunger was used to dispense the entire sample into the sample well of a cassette for analysis in the Cholestech LDX device (Cholestech Corp., San Diego, CA, USA). Analytes from the Cholestech LDX were obtained using the Lipid Profile + Glucose Cassettes that included glucose, total cholesterol (TC), high-density lipoprotein cholesterol (HDL-C), low-density lipoprotein cholesterol (LDL-C), and triglycerides (TG), and were previously found to be comparable to laboratory blood draws utilizing the National Health System (NHS) [15,16,17,18]. Glucose and TG were not used in the EFA because of their sensitivity to fasting vs. fed states. To measure hemoglobin A1c (A1c), 0.5 mcg of blood was collected into another capillary tube that was placed into another cassette and into the Alere Affinion analyzer (Alere Inc., Waltham, MA, USA) [18].

Blood pressure: Blood pressure was measured using a Welch Allyn 71WT-B Connex Spot blood pressure monitor (Hill-Rom Holdings, Inc., Skaneateles Falls, NY, USA). The participant was instructed to sit for 5–10 min prior to testing. The appropriately sized cuff (regular, long) was placed on the participant’s left bicep and measured in triplicate and averaged.

### 2.4. Body Size Measures

InBody S10 (InBody^®^, Cerritos, CA, USA) body composition analyzer: This device was used to measure BF% in trials NCT04014296, NCT04745572, and NCT04392284. Participants were instructed to remove their socks, shoes, excess clothing, and jewelry. They were asked to lay supine on a clinic bed for 10 min prior to measurement to allow for body fluid to equilibrate. Their arms and legs were spread apart to avoid contact with the trunk region. Touch-type electrodes were clipped to participants’ thumbs, middle fingers, and ankles after these areas were cleansed with the manufacturer’s body tissue wipe. Demographic parameters including weight, height, age, and sex were used for computing analyses.

Dual-energy X-ray absorptiometry (DXA): A whole-body DXA scan was completed using the GE Lunar Prodigy Primo (encore software version 15.10.046, GE Healthcare, Chicago, IL, USA) in clinical trial, NCT03832933, and used to measure BF%. A quality assurance assessment was conducted prior to use. Participants were instructed to remove jewelry and wear clothing without zippers, wires, or metal accessories and positioned in a supine position under the scanning arm according to the manufacturer’s instructions. Good agreement has been found between InBody S10 and DXA [19,20,21], especially when supine (intraclass correlation coefficient = 0.94) [22].

Waist circumference: Waist circumference was measured at the boarder of the iliac crest in accordance with National Institutes of Health [23]. Two measures were obtained consecutively, and the average was used for analyses.

Body mass index (BMI): To calculate BMI (kg/m^2^), weight was obtained from a platform scale (either DETECTO BRW1000, DETECTO, Webb City, MO, or Health O Meter Professional Scales, McCook, IL, USA) with an accuracy ±0.1 kg while they were dressed in lightweight clothing and without shoes. For each trial, the same scale was used at baseline and follow-up testing. Height was measured using a stadiometer to the nearest 0.5 cm.

### 2.5. Statistical Analysis

The EFA was conducted in *n*= 249 people to determine the factor structure of metabolic and body size measures. An unweighted least squares (ULS) extraction method was used because it is recommended when only few factors are expected to be retained and if communalities are high (>0.6). To allow variables to be correlated, an oblique rotation method (direct oblimin) with Kaiser normalization was used. Nine items were included in the EFA. Model fit statistics, communalities, and reproduced correlation residuals were analyzed and interpreted. Factors with eigenvalues > 1 (Kaiser’s criterion) were selected for retention and scree plots were analyzed to confirm. Items with coefficients < 0.6 were considered for removal to ensure the most parsimonious model was produced with moderate-to-high item-to-factor correlations. To measure scale reliability, Cronbach’s alpha was analyzed and values > 0.60 were considered satisfactory [24]. Internal consistency was measured by item total correlations and values > 0.20 were considered satisfactory [25]. Cases were excluded listwise to handle missing data. Statistical analyses were performed using IBM SPSS Statistics software (version 29 for Windows).

## 3. Results

### 3.1. Exploratory Factor Analysis

**Figure 1:**
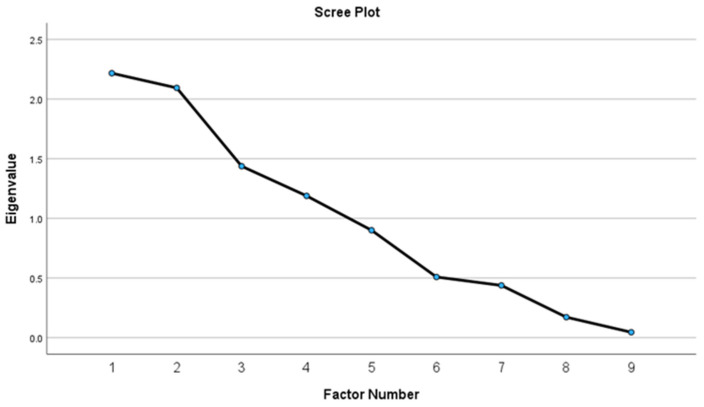
Scree plot of initial four-factor solution.

**Figure 2:**
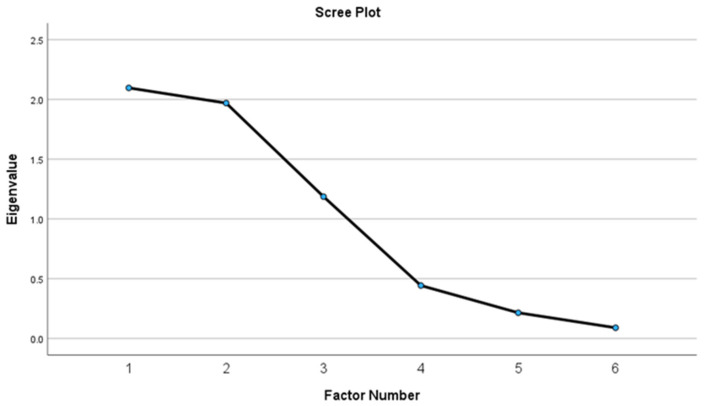
Scree plot of final three-factor solution.

The EFA included nine items from *n* = 249 adults (55.7 ± 11.3 years; 74.7% female) with obesity (BMI 38.5 ± 6.7 kg/m^2^). See Table 3 for descriptive statistics and Table 4 for model fit statistics. The scree plot produced after the first round of dimension reduction suggested there were four factors with eigenvalues > 1.0 that explained 77.1% of the variance (Figure 1). Items that did not factor load (coefficients < 0.60) were removed one at a time and replaced back into the model so that every combination of items was tested. Items were removed if their exclusion produced a more parsimonious model with greater explained variance. The following items were removed in order: A1c, BF%, and HDL-C. The final factor solution included three factors explaining 87.6% of the variance as shown in Figure 2 and Table 5. LDL-C and TC loaded on factor one, BMI and WC loaded on factor two, and SBP and DBP loaded on factor three.

### 3.2. Item Total Correlations

Internal consistency (α) and item/total correlations were calculated to analyze the final scales for each factor (Table 6). The range of item/total correlations were satisfactory for the lipid factor (0.91), the body size factor (0.79), and the blood pressure factor (0.56). Reliability (α) was also satisfactory for the lipid measures (0.95), the body size measures (0.74), and the blood pressure measures (0.67).

## 4. Discussion

The final EFA model revealed three dimensions: lipids, body size, and blood pressure. Results supported the hypothesis that measures of body size would factor load separately from measures of metabolic health, but the distinction of lipid and blood pressure measures of metabolic health was not anticipated. The model also demonstrated acceptable internal consistency of the scales and item/total correlations. These findings support that measures of body size are distinct from CMD risk factors and lend credence to HAES^®^ approaches that advocate for weight-neutral interventions for improving health and well-being in people with obesity.

An EFA is a data-driven approach to identify unobservable constructs and determine the dimensionality of two or more variables. It recognizes patterns in the way that items of a dataset group (e.g., covary) together. The items that group together the strongest are considered to have an underlying construct that interconnects them. Items that do not possess patterns in their covariances are loaded onto separate factors and considered to be multidimensional. Our final EFA model showed that CMD risk factors were multidimensional from measures of body size, indicating the presence of latent constructs driving their distinction.

The constructs of body size and metabolic disease are often conflated because it is well known that there is strong association between the two [3,26]. However, there are cases where obesity can exist without CMD and vice versa. An example is the metabolically obesity healthy (MHO) phenotype in which someone with obesity does not have metabolic disease (i.e., insulin resistance, dyslipidemia, or hypertension) [27]. It is also possible to have cardiometabolic diseases like type 2 diabetes [28] or cardiovascular disease [29,30] without having obesity, also sometimes called the metabolically unhealthy normal weight phenotype [31]. It is obvious that in many cases obesity and CMD coexist, but results of the present study suggest they may be driven by distinct factors. Therefore, each distinct factor could be intervened on separately as part of a patient-centered obesity treatment regimen. Importantly, the well-known connection between obesity and CMD should not be minimized, but rather the distinction between their constructs may present opportunities for other treatment avenues. For example, one could prefer to manage their metabolic health rather than their body size. Physical activity could help prevent CMD with or without weight loss; however, changes in body size/weight are likely to cooccur [32,33].

Obesity is driven by a diverse set of behavioral, physiological, and environmental factors that vary considerably across individuals and populations [34]. Interindividual variability among these driving factors could explain the identified latent constructs driving the distinction between body size and metabolic health. Since obesity’s development and expression is multifactorial, treatment options should also be diverse. Although weight loss should still be included in an obesity treatment paradigm, a constant focus on it may also promote unhealthy attitudes and behaviors such as body dissatisfaction, eating disorders, low self-esteem, and disinterest in physical activity. Therefore, HAES^®^-based weight-neutral interventions could be used to expand the focus from just weight loss to include more holistic obesity treatment regimens.

A strength of this study is the sample size, which provided a 27:1 subject to item ratio. Although the sample size required for an EFA is not standardized, a ratio of at least 20:1 is generally acceptable [35]. A potential limitation of this research is that our sample consisted mostly of older adult females (mean age 55.7 ± 11.3, 74.7% female) and people with obesity. Therefore, generalizability to men, younger populations, and/or those with MHO or metabolically unhealthy normal weight phenotypes is weaker. Additionally, methods used to collect blood (POC vs. laboratory-drawn blood) and BF% (DXA vs. BIA) varied across studies, but previous research has reported the body composition methods used in the included studies demonstrated suitable agreement [22]. Future research requires a confirmatory factor analysis (CFA) to confirm these results. Additional CFAs (and possibly additional EFAs) will need to be conducted in more diverse populations.

## 5. Conclusions

Measures of body size factor loaded separately from measures of metabolic health among people with obesity who were entering a weight loss trial, and metabolic health was further split into lipid- and blood pressure-focused factors. These results provide opportunities for both weight-focused (i.e., weight loss) and weight-neutral interventions to improve overall health and well-being [36]. Future research requires a CFA and investigation into additional populations with greater age, sex/gender, and body size diversity.

## Figures and Tables

**Table 1:** Descriptions of studies used in present analysis.

Study	NCT04014296 ^1^	NCT04745572 ^1^	NCT04392284 ^1^	NCT03832933 [14]
Purpose	To compare the separate and combined effects of a high-protein diet and resistance training on retention of fat-free mass during weight loss in older adults.	To compare a high and reduced carbohydrate diet with or without individual counseling for time-restricted eating or exercise.	To investigate the feasibility of an adaptive biobehavioral intervention for improving insulin sensitivity among patients with Stage 1 obesity.	To compare a high-protein diet with ≥4 weekly servings of lean beef and a normal-protein diet without any red meat for weight loss, body composition changes, and glucose control in individuals with T2D.
Study design	SMART	SMART	SMART	RT
Original study *N*	89	83	40	106
*n* for present analysis	52	53	40	104
Age	≥50 years old	18–75 years old	18–65 years old	≥18 years old
BMI	≥30 kg/m^2^	27 kg/m^2^	≥27 kg/m^2^	≥27 kg/m^2^
Main inclusion criteria	Must be postmenopausal if female (≥1 year since last menstrual period).	Must have prediabetes (A1c ≥ 5.7% and/or fasting glucose ≥ 100 mg/dL).	Must have one or more mild-to-moderate obesity-related complication such as prediabetes, type 2 diabetes, metabolic syndrome, dyslipidemia, hypertension, non-alcoholic fatty liver disease, etc.	Must have a diagnosis of T2D within the previous 6 years by either a documented physician diagnosis, use of antidiabetic medication, or fasting glucose ≥ 126 mg/dL, and/or HbA1c ≥ 6.5%.
Additional criteria	Participants could not have a pacemaker or other battery-operated implant, no use or stable use (≥3 months on same dosage) of medications affecting body weight, and were not taking insulin.	Participants could not have a pacemaker or other battery-operated implant, no use or stable use (≥3 months on same dosage) of medications affecting body weight, and were not taking insulin.	Participants could not have a pacemaker or other battery-operated implant, no use or stable use (≥3 months on same dosage) of medications affecting body weight, and were not taking insulin.	No use or stable use (≥3 months on same dosage) of medications affecting body weight and were not taking insulin.

^1^ Data from studies NCT04014296, NCT04745572, and NCT0439228 are not published at the time of this manuscript so references cannot be provided. Abbreviations: BMI—body mass index; SMART—sequential multiple assignment randomized trial; RT—randomized trial.

**Table 2:** Participant characteristics (*n* = 249).

Characteristic	Mean ± SD or *n* (%)				
	Present Study	NCT04014296	NCT04745572	NCT04392284	NCT03832933 [14]
Age (years)	55.7 ± 11.3	62.0 ± 7.3	52.9 ± 12.7	53.1 ± 12.2	54.8 ± 10.9
Race/Ethnicity					
Non-Hispanic White	123 (49.2)	31 (59.6)	16 (30.2)	10 (25.0)	66 (63.5)
Non-Hispanic Black	111 (44.6)	19 (36.5)	34 (64.2)	29 (72.5)	29 (27.9)
Asian	6 (2.4)		1 (1.9)	1 (2.5)	4 (3.8)
Native Hawaiian or Pacific Islander	1 (0.4)				1 (1.0)
Other	8 (3.2)	2 (3.8)	2 (3.8)		4 (3.8)
Sex					
Male	63 (25.3)	23 (44.2)	8 (15.1)	7 (17.5)	25 (24.0)
Female	186 (74.7)	29 (55.8)	45 (84.9)	33 (82.5)	79 (76.0)
Height (cm)	166.9 ± 9.1	170.1 ± 9.9	164.3 ± 7.1		166.4 ± 9.7
Weight (kg)	107.8 ± 23.1	111.8 ± 19.5	108.6 ± 24.9	106.0 ± 28.2	106.1 ± 21.7
BMI (kg/m^2^)	38.5 ± 6.7	38.5 ± 5.0	39.6 ± 7.6	37.6 ± 8.0	38.3 ± 6.4
25th percentile	33.4	34.2	33.9	30.5	33.3
50th percentile	37.3	38.2	39.4	35.2	37.0
75th percentile	43.3	41.9	44.1	43.3	43.2
WC (cm)	117.8 ± 15.3	121.7 ± 13.1	118.4 ± 17.6	114.4 ± 18.8	117.0 ± 13.3

Characteristics are shown for participants at baseline. Abbreviations: SD—standard deviation; BMI—body mass index; WC—waist circumference.

**Table 3:** Descriptive statistics.

*n* = 249	Mean	SD
SBP	134.3	15.4
DBP	85.2	9.7
A1c	6.4	1.1
HDL-C	49.7	13.4
LDL-C	103.1	34.8
TC	172.7	39.9
BMI (kg/m^2^)	38.5	6.7
BF%	45.8	9.0
WC (cm)	117.8	15.3

Abbreviations: SD—standard deviation; SBP—systolic blood pressure; DBP—diastolic blood pressure; A1c—hemoglobin A1c; HDL—high-density lipoprotein cholesterol; LDL—low-density lipoprotein cholesterol; TC—total cholesterol; BMI—body mass index; BF%—body fat percent; WC—waist circumference.

**Table 4:** Model fit statistics.

Kaiser–Meyer–Olkin Measure of Sampling Adequacy	0.528
Approx. Chi-Square	781.805
*df*	15
Sig.	<0.001

Abbreviations: Approx.—approximately; *df*—degrees of freedom; sig.—significance.

**Table 5:** Pattern matrix of final three-factor solution.

Factor	1	2	3
SBP			.864
DBP			.643
LDL	.991		
TC	.916		
BMI		.805	
WC		.974	

Extraction method: unweighted least squares; rotation method: oblimin with Kaiser normalization; rotation converged in 5 iterations. Abbreviations: SBP—systolic blood pressure; DBP—diastolic blood pressure; LDL—low-density lipoprotein cholesterol; TC—total cholesterol; BMI—body mass index; WC—waist circumference.

**Table 6:** Corrected item/total correlations and alpha of factors if item were deleted.

Factor	Corrected Item/Total Correlation	Cronbach’s Alpha
LDL-C	0.91	0.95
TC		
BMI	0.79	0.74
WC		
SBP	0.56	0.67
DBP		

## Data Availability

The original data presented in the study are openly available in PubMed Central at https://www.ncbi.nlm.nih.gov/pmc/ (accessed on 2 August 2024).
